# Mucocutaneous *Leishmania tropica* infection in a dog from a human cutaneous leishmaniasis focus

**DOI:** 10.1186/1756-3305-7-118

**Published:** 2014-03-24

**Authors:** Gad Baneth, Doni Zivotofsky, Yaarit Nachum-Biala, Daniel Yasur-Landau, Ana-Maria Botero

**Affiliations:** 1 School of Veterinary Medicine, Hebrew University, P.O. Box 12, Rehovot 76100, Israel; 2 Maale Adumim Veterinary Clinic, Maale Adumim, Israel; 3 Pathovet LTD, Yehosa Ben Hanania 81, Rehovot 76391, Israel

**Keywords:** *Leishmania tropica*, Mucocutaneous leishmaniasis, Dog, Israel, Allopurinol

## Abstract

**Background:**

*Leishmania tropica* is a causative agent of cutaneous leishmanaisis in the Middle East, North Africa and parts of southeastern Europe. Although transmission of *L. tropica* has been reported as anthroponotic, in Israel it was found to have a zoonotic pattern.

**Findings:**

A one year old male Pekingese dog from Maale Adumim, a focus of *L. tropica* human cutaneous leishmaniasis near Jerusalem, was presented by its owner with a large proliferative red mucocutaneous lesion on the lip between the mouth and nose. Physical examination and a biochemistry panel were normal and a complete blood count showed mild leukocytosis with lymphocytosis and eosinophilia. A biopsy of the lesion was suggestive of the presence of *Leishmania* organisms. Serology for *Leishmania* sp. by ELISA was positive and an aspirate from the lesion showed a large number of *Leishmania* amastigotes. ITS1-HRM-PCR of the lesion was positive and sequencing indicated that infection was caused by *L. tropica,* which was also cultured from the lesion*.* Blood PCR was negative. The dog responded well to allopurinol treatment and its lesion shrunk considerably within one month of therapy and healed after two months.

**Conclusions:**

Only a few cases of dog infection with *L. tropica* have been described to date. They were reported from Morocco and Iran and involved infection of visceral organs. This is the first report of focal mucocutaneous *L. tropica* infection in a dog and its response to anti-leishmanial treatment. Domestic and wild canines should be evaluated for being possible animal reservoirs for human *L. tropica* infection in endemic areas or merely accidental hosts.

## Findings

**Figure 1:**
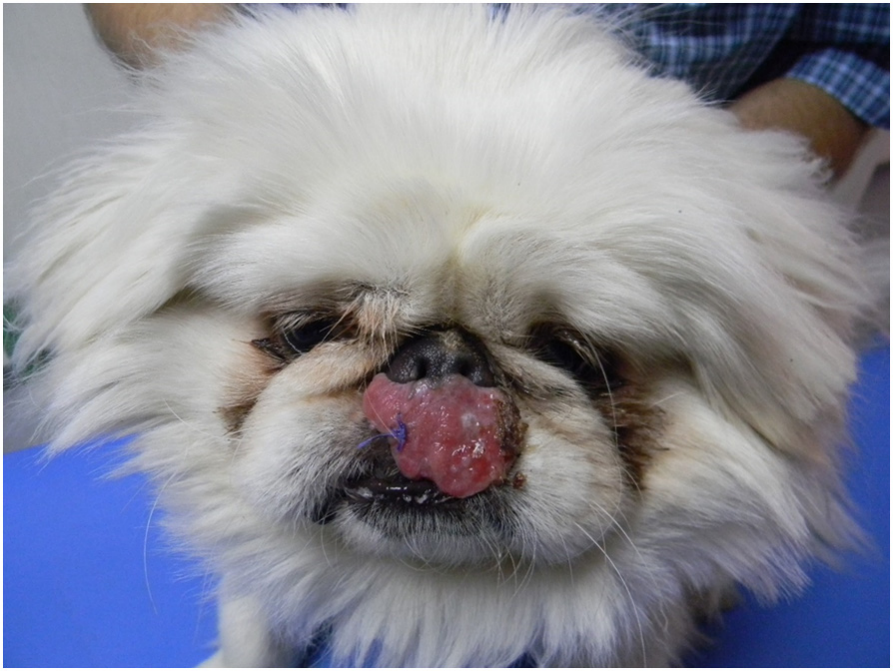
**Mucocutaneous *Leishmania tropica *in the dog.** Dog showing mucocutaneous lesion caused by *Leishmania tropica* before treatment.

**Figure 2:**
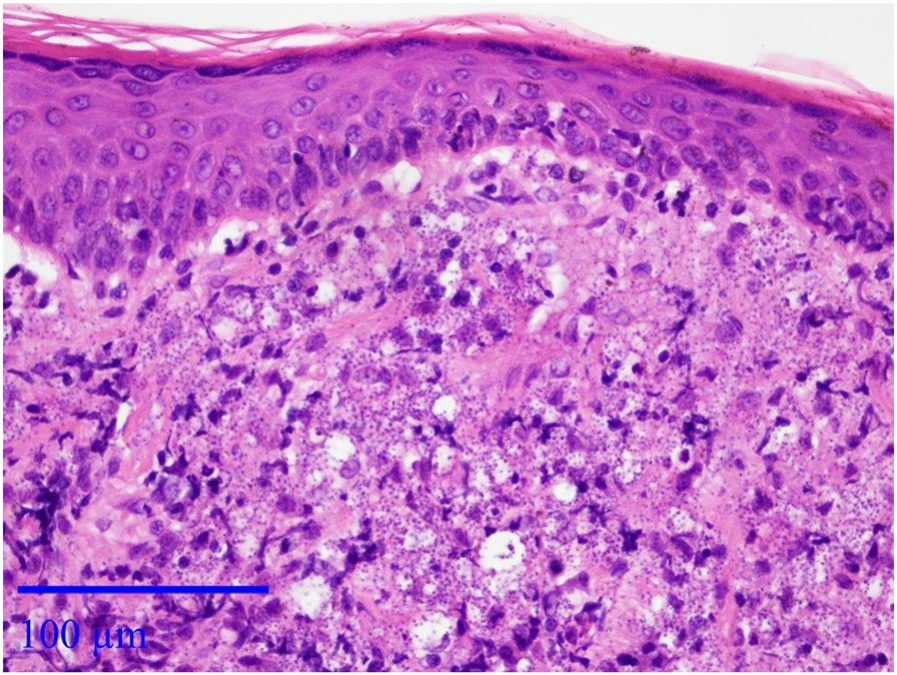
**Histological section, mucocutaneous lesion.** There is moderate epidermal hyperplasia and diffuse superficial dermal infiltration with numerous macrophages laden with numerous *Leishmania* organisms. *Leishmania* are also observed in the extracellular space. Hematoxylin & Eosin stain, Bar = 100 μm.

**Figure 3:**
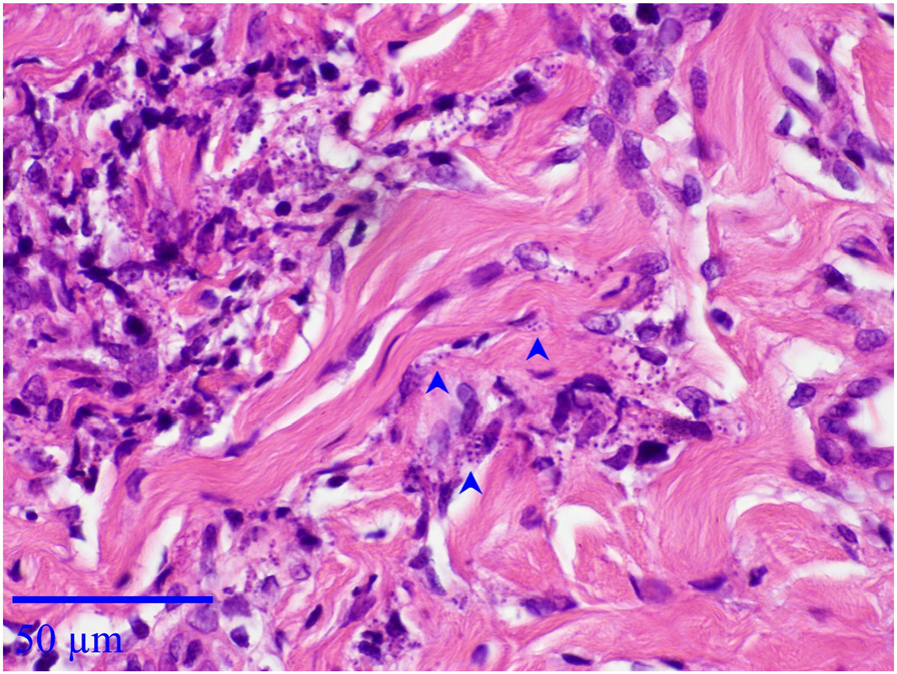
**Histological section, mucocutaneous lesion, deep dermis.***Leishmania* amastigotes are both intrahistiocytic and extracellular. Note presence of *Leishmania* organisms in fibroblasts (arrowheads). Hematoxylin & Eosin stain, Bar = 50 μm.

A one year old male Pekingese dog from Maale Adumim, a focus of *Leishmania tropica* human cutaneous leishmaniasis near Jerusalem, Israel, was presented by its owner to a local veterinary clinic with a large proliferative red mucocutaneous lesion on the upper lip extending all the way between the nose and mouth (Figure 1). Physical examination was otherwise normal and the dog was initially treated with the antibiotic cephalexin at 20 mg/kg orally every 12 hours for 10 days and with the antifungal itraconazole at 5 mg/kg/day orally for 10 days for suspected bacterial dermatitis or fungal infection. After no improvement was observed, a full thickness biopsy of the lesion was taken under short general anesthesia and submitted for histological evaluation. Microscopically, the skin had extensive epidermal ulceration with underlying heavy dermal infiltration of numerous reactive macrophages interspersed with neutrophils and aggregations of plasma cells. Macrophages contained numerous basophilic oval organisms, measuring approximately 1 to 2 μm, interpreted as *Leishmania* amastigotes (Figures 2 and 3). Amastigotes were also present in fibroblasts as previously reported for *Leishmania infantum*[1] (Figure 3). Following the presumptive diagnosis of leishmaniasis, a complete blood count (CBC) and serum biochemistry panel were taken, as well as serology for *L. infantum* by ELISA as previously described [2] and blood for PCR using the ITS1-PCR- high resolution melt (HRM) analysis [3]. The lesion was aspirated for cytology, ITS1-PCR-HRM, and parasite culture in NNN slants overlaid with Schneider’s *Drosophila* medium as previously described [2].

**Figure 4:**
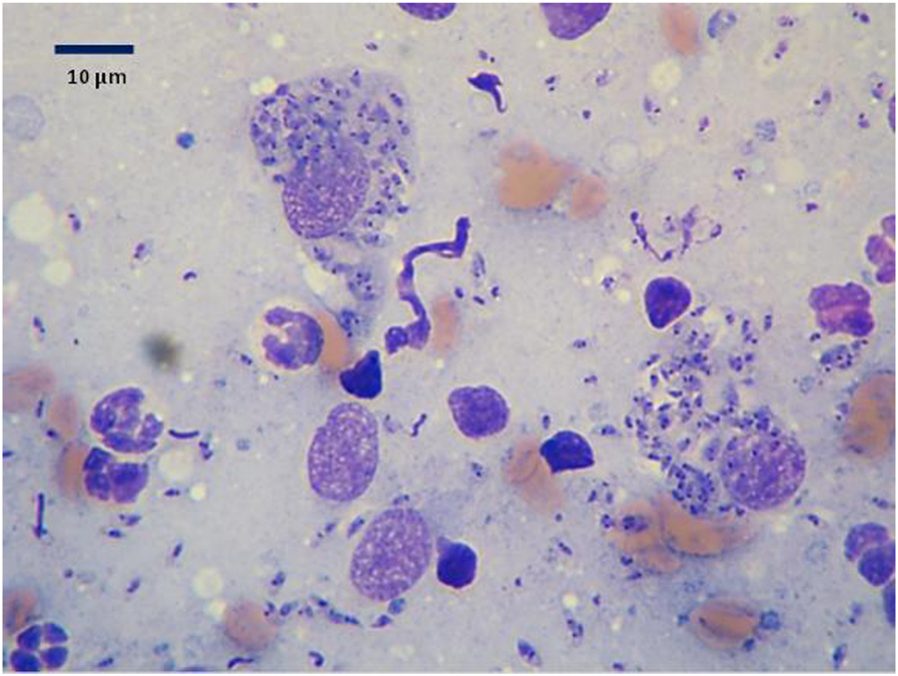
**Cytology of lesion.** Cytology of aspirate from the mucocutaneous lesion stained by May Grunwald Giemsa showing numerous intracellular *Leishmania* amastigotes and some extracellular amastigotes persumably freed from the host cell during the aspiration.

A blood count showed a leukocytosis (17.9 × 10^3^ leukocytes/μl; reference 5.2-13.9 × 10^3^) comprised of lymphocytosis (7.87 × 10^3^ lymphocytes/μl; reference 1.3-4.1 × 10^3^) and eosinophilia (1.61 × 10^3^ eosinophils/μl; reference 0–0.6 × 10^3^), with no anemia or thrombocytopenia. The biochemistry panel was normal with no hyperglobulinemia or hypoalbuminemia typical of canine *L. infantum* infection. ELISA serology was positive with an optical density (O.D.) of 1.88 (cut off 0.6 O.D.). Cytology of the lesion aspirate showed a large number of *Leishmania* amastigotes in macrophages and also outside cells presumably freed from breaking host cells during the aspiration (Figure 4). The culture taken by needle aspiration was positive for *Leishmania* promstigotes and ITS1-PCR-HRM was negative in blood and positive from the culture with an HRM curve compatible with *L. tropica*[3]. Sequencing of a 253 bp ITS1 PCR DNA product was carried out at the Hebrew University and the obtained sequence was deposited in GenBank [KF974365] and found by BLAST analysis (http://www.ncbi.nlm.nih.gov/BLAST) to be 100% identical to the closest match, *L. tropica* from Iran [KC505439], with 99% coverage. An additional 400 bp ITS1 fragment was amplified using primers ITS1F and ITS2R4 essentially as previously described [4] with some modification in the thermal profile, e.g. initial denaturation at 95°C for 5 min, followed by 37 cycles each consisting of: 30 s at 95°C (denaturation), 30 s at 58°C (annealing), and 90s at 72°C (extension). After the last cycle, the extension step was continued for a further 5 min. The 400 bp ITS1 fragment was sequenced and submitted to GenBank [KJ010813]. The *L. tropica* isolate from the dog’s lesion was maintained in culture and frozen down as strain LRC-L1647.

**Figure 5:**
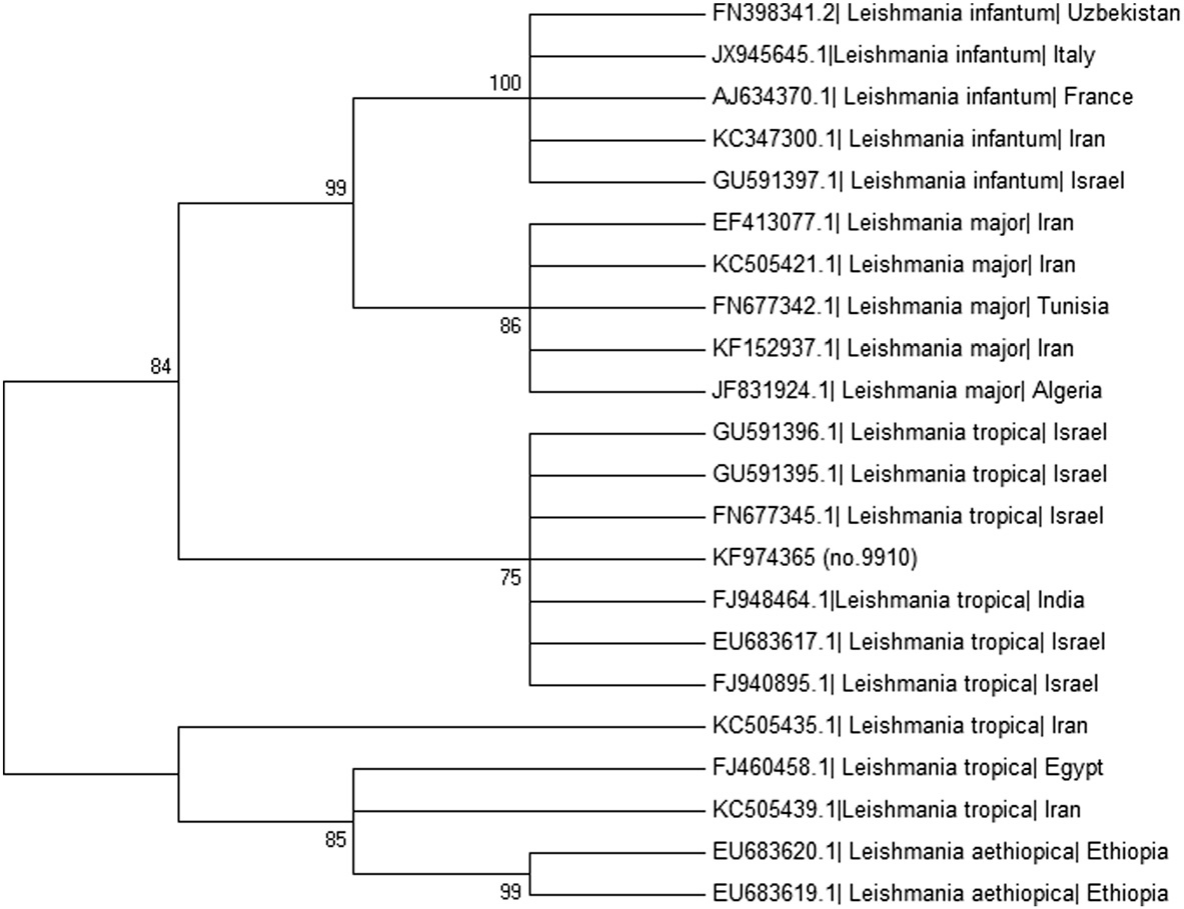
**Phylogram.** A Maximum Likelihood phylogram comparing 253 bp ITS1 rRNA DNA *Leishmania* sequences from the Israeli dog *L. tropica* strain [KF974365] with other *L. tropica* strains and Old World *Leishmania* spp. The GenBank accession numbers, *Leishmania* sp. and country of origin are included for each sequence.

Phylograms of the 253 and 400 bp *L. tropica* ITS1 fragments [KF974365 and KJ010813] from the dog were constructed to compare these sequences to other *L. tropica* strains and additional *Leishmania* spp. present in GenBank. Sequences were analyzed using the MEGA version 5.1 (http://www.megasoftware.net) and phylograms were constructed using the Maximum likelihood algorithm with the Tamura-Nei model. Bootstrap replicates were performed to estimate the node reliability, and values were obtained from 500 randomly selected samples of the aligned sequence data (Figure 5 and Additional file 1: Figure S1). The phylograms indicated that the dog’s *L. tropica* sequences clustered together with other *L. tropica* strains from Israel and other Asian countries, separately from a second group of *L. tropica* strains which clustered together with *L. aethiopica*, and also away from *L. major* and *L. infantum*.

**Figure 6:**
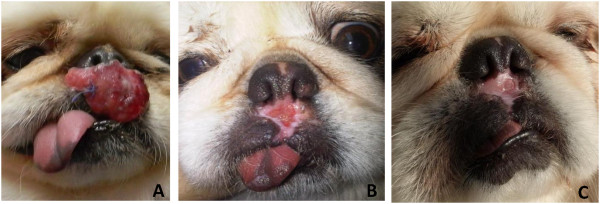
**Treatment reposne.** Response of lesion to allopurinol treatment (10 mg/kg every 12 hours). **A**- before treatment; **B** – 4 weeks after begining of treatement; **C**– 8 weeks after begining of treatment.

The dog was treated with allopurinol at 10 mg/kg every 12 hours as recommended for dogs infected with *L. infantum*[5]. One month after the initiation of treatment the dog’s lesion was in a progressive state of healing, had considerably shrunk in size and appeared to be scarring. Two months after treatment initiation the lesion had almost disappeared and was replaced by depigmented skin and a small round scar (Figure 6). The owners reported that the dog was active and in good health. Repeat CBC and biochemistry panel were within normal limits except for a persisting lymphocytosis of 5.87 lymphocytes/μl and eosinophilia of 1.28 × 10^3^/μl, which had decreased from the previous testing, in addition the ELISA serology O.D. decreased to 0.38 from 1.88.

Ma’ale Adumim, the city where the dog described here lives, is a major emerging focus of human cutaneous leishmaniasis where 54 and 73 *L. tropica* human cases were recorded during 2004 and 2005, respectively. These numbers reflect a sharp increase in the annual incidence from 2 cases per 100,000 residents from 1999 until 2003 to 214 per 100,000 residents in 2004 [6].

*Leishmania tropica* is an important causative agent of cutaneous leishmaniasis in the Old World including the Middle East, North Africa, Central Asia and some parts of southern Europe. It has also been described as a rare cause of human visceral leishmaniasis [7]. Although cutaneous leishmaniasis caused by *L. tropica* is usually considered an anthroponotic infection transmitted between people directly by phlebotomine sand flies without the involvement of an animal reservoir [8], in Israel, Jordan and the Palestinian Authority it is a zoonosis with the rock hyrax (*Procavia capensis*) as a main reservoir host [9,10]. Golden jackals (*Canis aureus*) and red foxes (*Vulpes vulpes*) have also been found to be infected with *L. tropica* in Israel and assumed to have a role in transmission of the infection between distant locations, but clinical signs of infection in these wild canids have not been detected [11].

In the domestic dog, *L. tropica* infection has been described in only a few cases reported from Morocco and Iran where infection was mostly described to involve visceral organs [12-16]. While some surveys described the detection of parasite infection from dog organs by culture or PCR without much detail on the manifestations of disease [14,16], reports from Morocco described two dogs infected with *L. tropica* with clinical manifestations similar to those found in canine *L. infantum* infection including generalized lymphadenomegaly, onychogryphosis, alopecia, keratoconjunctivitis, and also glomerulonephritis in one case [12,13]. A report from northwestern Iran also described *L. tropica* in a dog with cutaneous and visceral involvement comparable to canine *L. infantum* infection [17]. However, an earlier report from Morocco described seven dogs infected with *L. tropica* that had only dermal manifestations consisting of small facial papules without lymphadenomegaly or splenomegaly [18]. In addition, a five month old pup with multiple lesions adjacent to the eyes, lips and jaw described as mucocutaneous, as well as generalized lymphadenomegaly and visceral infection of the spleen and liver, was reported from central Iran [15].

The dog described in the current report was admitted with a distinctive presentation of a single large proliferative mucocutaneous lesion that contained a large amount of *L. tropica* amastigotes, which is different from the typical dermal manifestations of canine *L. infantum* infection and also from the descriptions of visceralizing *L. tropica* in dogs. The dog did not present with any manifestations of involvement of internal organs or hematological and serum biochemical abnormalities common in visceral infection such as anemia, hyperglobulinemia, or hypoalbuminemia, and there was no indication that infection had progressed beyond the skin as the blood PCR was negative, although such progression cannot be ruled out. The lymphocytosis and eosinophilia found by CBC are not common findings in canine leishmaniasis and could be explained by some unique inflammatory response with an eosinohilic and lymphocytic component. The seropositivity to *L. infantum* antigen was also reported in other cases of canine *L. tropica* infection [12,13] and is not surprising as there is a strong serological cross-reactivity between different *Leishmania* species. None of the dogs described with *L. tropica* infection previously have been treated with anti-leishmanial drugs. The excellent initial response to allopurinol treatment and the healing of the dog’s lesion indicate that allopurinol used as the major drug against canine *L. infantum* infection, is also effective against canine *L. tropica* infections.

## Conclusions

This is the first report of focal mucocutaneous *L. tropica* infection in a dog and its response to anti-leishmanial treatment. Domestic and wild canine infection with *L. tropica* may be more prevalent in areas of endemic human *L. tropica* cutaneous leishmaniasis than currently recognized, and canines should be evaluated as possible additional reservoirs for human infection.

## Competing interests

The authors declare that they have no competing interests.

## Authors’ contributions

GB collected the data, sampled the dog and wrote the manuscript; DZ is the veterinarian who saw the dog, biopsied it, administered treatment and continued to monitor its progress; YNB performed serology, PCR and DNA sequencing and performed the phylogenetic analyses; DYS isolated *L. tropica* and grew the parasite in culture; and AMB performed and interpreted the histopathology of the lesion. All authors read and approved the final version of the manuscript.

## Supplementary Material

Additional file 1: Figure S1 Phylogram of 400 bp ITS1 fragment. A Maximum Likelihood phylogram comparing 400 bp ITS1 rRNA DNA *Leishmania* sequences from the Israeli dog *L. tropica* strain [KJ010813] with other *L. tropica* strains and Old World *Leishmania* spp. The GenBank accession numbers, *Leishmania* sp. and country of origin are included for each sequence. Click here for file
